# Comparative Genotypes, Staphylococcal Cassette Chromosome mec (SCCmec) Genes and Antimicrobial Resistance amongst *Staphylococcus epidermidis* and *Staphylococcus haemolyticus* Isolates from Infections in Humans and Companion Animals

**DOI:** 10.1371/journal.pone.0138079

**Published:** 2015-09-17

**Authors:** Brenda A. McManus, David C. Coleman, Emily C. Deasy, Gráinne I. Brennan, Brian O’ Connell, Stefan Monecke, Ralf Ehricht, Bernadette Leggett, Nola Leonard, Anna C. Shore

**Affiliations:** 1 Microbiology Research Unit, Dublin Dental University Hospital, University of Dublin, Trinity College Dublin, Dublin, Ireland; 2 National MRSA Reference Laboratory, St. James’s Hospital, James’s St., Dublin 8, Ireland; 3 Department of Clinical Microbiology, School of Medicine, Trinity College Dublin, St. James’s Hospital, Dublin 8, Ireland; 4 Alere Technologies GmbH, Jena, Germany; 5 Institute for Medical Microbiology and Hygiene, Medizinische Fakultät "Carl Gustav Carus", Technische Universität Dresden, Dresden, Germany; 6 InfectoGnostics Research Campus, Jena, Germany; 7 Pathobiology, School of Veterinary Medicine, University College Dublin, Belfield, Dublin 4, Ireland; Rockefeller University, UNITED STATES

## Abstract

This study compares the characteristics of *Staphylococcus epidermidis* (SE) and *Staphylococcus haemolyticus* (SH) isolates from epidemiologically unrelated infections in humans (Hu) (28 SE-Hu; 8 SH-Hu) and companion animals (CpA) (12 SE-CpA; 13 SH-CpA). All isolates underwent antimicrobial susceptibility testing, multilocus sequence typing and DNA microarray profiling to detect antimicrobial resistance and SCC*mec*-associated genes. All methicillin-resistant (MR) isolates (33/40 SE, 20/21 SH) underwent *dru* and *mecA* allele typing. Isolates were predominantly assigned to sequence types (STs) within a single clonal complex (CC2, SE, 84.8%; CC1, SH, 95.2%). SCC*mec* IV predominated among MRSE with ST2-MRSE-IVc common to both Hu (40.9%) and CpA (54.5%). Identical *mecA* alleles and nontypeable *dru* types (dts) were identified in one ST2-MRSE-IVc Hu and CpA isolate, however, all *mecA* alleles and 2/4 dts detected among 18 ST2-MRSE-IVc isolates were closely related, sharing >96.5% DNA sequence homology. Although only one ST-SCC*mec* type combination (ST1 with a non-typeable [NT] SCC*mec* NT9 [class C *mec* and *ccrB4*]) was common to four MRSH-Hu and one MRSH-CpA, all MRSH isolates were closely related based on similar STs, SCC*mec* genes (V/V_T_ or components thereof), *mecA* alleles and dts. Overall, 39.6% of MR isolates harbored NT SCC*mec* elements, and ACME was more common amongst MRSE and CpA isolates. Multidrug resistance (MDR) was detected among 96.7% of isolates but they differed in the prevalence of specific macrolide, aminoglycoside and trimethoprim resistance genes amongst SE and SH isolates. Ciprofloxacin, rifampicin, chloramphenicol [*fexA*, *cat-pC221*], tetracycline [*tet*(K)], aminoglycosides [*aadD*, *aphA3*] and fusidic acid [*fusB*] resistance was significantly more common amongst CpA isolates. SE and SH isolates causing infections in Hu and CpA hosts belong predominantly to STs within a single lineage, harboring similar but variable SCC*mec* genes, *mecA* alleles and dts. Host and staphylococcal species-specific characteristics were identified in relation to antimicrobial resistance genes and phenotypes, SCC*mec* and ACME.

## Introduction

Two clinically relevant coagulase-negative staphylococcal (CoNS) species, S*taphylococcus epidermidis* and *Staphylococcus haemolyticus*, are among the leading causes of nosocomial infections in humans, particularly in neonates, immunocompromised patients and patients with indwelling and implanted devices [1, 2]. A number of comparative population studies of CoNS species recovered from nasal swabs of domesticated animals, livestock and associated farmers and veterinary personnel [3–5] have indicated that CoNS may be transmitted between these hosts in close contact. To date, detailed comparative population analyses of specific CoNS species recovered from infections in humans and companion animals are lacking.

Methicillin resistance (MR) is more common among *S*. *epidermidis* and *S*. *haemolyticus* isolates from both animals and humans compared to *Staphylococcus aureus* [6–8]. These CoNS are a reservoir of staphylococcal cassette chromosome (SCC) elements for *S*. *aureus*, including SCC harboring the MR gene *mec* (SCC*mec*) and the SCC-like arginine catabolic mobile element (ACME) [9, 10] which facilities staphylococcal colonization of human skin [11, 12]. Several studies have indicated that a great diversity of SCC*mec* and ACME-*arc* associated genes exist among CoNS, and that particular CoNS species may be a reservoir for specific SCC*mec* elements or genes [13]. Such CoNS often carry non-typeable SCC*mec* elements with novel cassette chromosome recombinase (*ccr*) and *mec* gene complexes, or combinations of these genes, yet unidentified in methicillin resistant *S*. *aureus* (MRSA). These combinations may give rise to new SCC*mec* elements in MRSA [10, 13–15]. The direct-repeat unit (*dru*) region within the SCC*mec* element has proved useful for tracking the epidemiological spread of different SCC*mec* elements as well as for further discriminating MRSA [16].

In addition to methicillin, resistance to other antimicrobial agents has also been reported to be more common among CoNS than in *S*. *aureus* [4, 17–20]. Many of these resistance genes in CoNS are located on mobile genetic elements (MGEs) and similar genes have been identified in *S*. *aureus* indicating the horizontal transfer of these genes among staphylococci [15, 21]. To date, only a few studies that examined the antimicrobial resistance patterns among staphylococci differentiated between individual CoNS species, making the prevalence of antimicrobial resistance among individual species difficult to ascertain [3, 4, 22]. Importantly, few studies have investigated the correlation of antimicrobial resistance phenotype and the presence of specific antimicrobial resistance genes in specific CoNS species [23–25]. Lastly, previous studies that investigated the antimicrobial susceptibility of CoNS all utilized different panels of antimicrobial agents to each other, making direct comparisons difficult [5, 20, 22, 26]. In this regard, direct comparison of the antimicrobial resistance phenotypes and associated resistance genes of specific CoNS species recovered from human and animal infections could be highly informative.

Coagulase-negative staphylococci are the third most commonly isolated pathogen from bloodstream infections among patients in Irish hospitals [27]. Despite this, little is known about the molecular epidemiology and population structure of specific CoNS species from patients in Irish hospitals. Although studies have shown similar MRSA strains among humans and companion animals in Ireland [28, 29], there are no published data regarding the epidemiology of CoNS here.

Pulsed-field gel electrophoresis (PFGE) has for many years been considered the gold standard for molecular typing of *S*. *epidermidis* and *S*. *haemolyticus* during outbreak investigations but is unsuitable for investigating the relatedness of isolates recovered over long periods of time [30, 31]. However, multilocus sequence typing (MLST) schemes have also been developed for investigating the population structures of these species [30, 32]. Despite the identification of a high level of genetic diversity and a large numbers of sequence types (STs) within the *S*. *epidermidis* population, isolates from human and animal hosts worldwide predominantly belong to clonal complex 2 (CC2) [22, 33]. The application of MLST to *S*. *haemolyticus* has had limited success; the only scheme developed revealed that the majority of 48 *S*. *haemolyticus* isolates investigated belonged to one main lineage, despite being recovered from disparate geographic locations around the world and over a long time period. However, this study included just four veterinary *S*. *haemolyticus* isolates, two of which were closely related to the human clinical isolates examined by MLST [32].

The potential of companion animals and humans to act as sources of staphylococcal infections for each other, as well as to provide a genetic reservoir for *S*. *aureus* means that it is essential that their population structure, antimicrobial resistance and molecular characteristics are better understood. The aim of this study was to compare the population structures, the prevalence of SCC*mec* genes, ACME and antimicrobial resistance phenotypes and associated resistance genes, among *S*. *haemolyticus* and *S*. *epidermidis* isolates recovered from unrelated infections in both humans and companion animals. The objective was to determine if the population structures of epidemiologically unrelated infection causing isolates of the two species are similar in both human and animal hosts in the absence of direct transmission. This study also investigated the contribution of *S*. *haemolyticus* and *S*. *epidermidis* to the staphylococcal gene pool with particular regard to SCC*mec*-associated and antimicrobial resistance genes.

## Methods

### Isolates

A total of 40 *S*. *epidermidis* (SE) isolates, 28 from humans (Hu) and 12 from companion animals (CpA), and 21 *S*. *haemolyticus* (SH) isolates, eight Hu and 13 CpA, were investigated (Table 1 and S1 Table). All Hu isolates were recovered from patients in two separate acute hospitals in Dublin, Ireland; eight SE-Hu isolates were associated with neurosurgical meningitis and were recovered from either external ventricular drains (EVDs) in patients with device-related meningitis or from non-EVD cerebrospinal fluid specimens taken by lumbar puncture between 2004 and 2006 [34]. The remaining 20 SE-Hu and the eight SH-Hu isolates were recovered from blood cultures of patients attending a separate acute hospital between 2010 and 2011. The 12 SE-CpA isolates examined were recovered from a cat (*n* = 1), dogs (*n* = 10) and a horse (*n* = 1). The 13 SH-CpA isolates were recovered from a cat (*n* = 1), dogs (*n* = 3) and horses (*n* = 9). These CpA isolates were recovered primarily in animals with wounds or infections attending a tertiary referral veterinary hospital in Dublin between 2004 and 2011 (S1 Table). Isolates were stored on commercially available cryobeads (Microbank, Pro-lab Diagnostics, Cheshire, UK) at -70°C.

**Table 1:** MLST clonal complexes and sequence types and SCC*mec*- associated genes detected in *S*. *epidermidis* and *S*. *haemolyticus* isolates recovered from infections in humans and companion animals.

MR phenotype & species [*n*]	CC	ST^a^[*n*]	SCC/SCC*mec* type/genes detected^b^[*n*]	*mecA* alleles^d^[*n*]	*dru* types[*n*]
**MRSE [22 Hu, 11 CpA]**	2-I	2 [9 Hu, 9 CpA]	IVc [Class B *mec* (*mecA*, *ΔmecR1*, *ugpQ*) *dcs* & *ccrAB2*] [6 Hu]	ABSA01000066 [6 Hu]	dt9bd [3 Hu]
					Non-typeable [2 Hu]
					dt10h [1Hu]
			IVc [Class B *mec* (*mecA*, *ΔmecR1*, *ugpQ*) *dcs* & *ccrAB2*] [6 CpA]	ABSA01000066 [2 CpA]	dt9g [1 CpA]
					Non-typeable [1 CpA]
				AY786579 [2 CpA]	dt9bd [1 CpA]
					dt5l [1 CpA]
				BA000018 [1 CpA]	Non-typeable [1 CpA]
				AB037671 [1 CpA]	Non-typeable [1 CpA]
			III [Class A *mec* (*mecA*, *mecR1*,*mecI*, *ugpQ*, *xylR*) *dcs* & *ccrAB3*] [2 Hu]	GU235984 [1 Hu]	dt7ah [1 Hu]
				EU929081 [1 Hu]	dt7ah [1 Hu]
			III [Class A *mec* (*mecA*, *mecR1*,*mecI*, *ugpQ*, *xylR*) *dcs* & *ccrAB3*] & ACME-*arc* [2 CpA]	ABSA01000066 [1 CpA]	dt9bn [1 CpA]
				AY786579 [1 CpA]	dt5l [1 CpA]
			NT6 [Class A *mec* (*mecA*, *mecI*, *mecR1*, *ugpQ*, *xylR*), *dcs* & *ccrAB3*, *ccrB4*, *ccrC*] [1 Hu]	BA000018 [1 Hu]	dt9a [1 Hu]
			NT8 [Class A *mec* (*mecA*, *ugpQ*, *mecI*, *mecR1*, *xylR*), *dcs*] & ACME-*arc* [1 CpA]	AY786579 [1 CpA]	dt8b [1 CpA]
	2-II	6 [1 Hu]	NT3 [Class B *mec* (*mecA*, *ΔmecR1*, *ugpQ*), *dcs*, *kdp & ccrAB2*, *ccrA3*, *ccrB4*] & ACME-*arc* [1 Hu]	AY786579 [1 Hu]	dt10ac [1 Hu]
	2-I	35 [2 Hu]	NT1 [Class B *mec* (*mecA*, *ΔmecR1*, *ugpQ)* & *ccrAB2*, *ccrC* (IVc)] & ACME-*arc* [1 Hu]	AY786579 [1 Hu]	dt10g [1 Hu]
			NT2 [Class B *mec* (*mecA*, *ΔmecR1*, *ugpQ*), *dcs* & *ccrAB2*, *ccrA1* (IVa)] & ACME-*arc* [1 Hu]	EU929081 [1 Hu]	dt9g [1 Hu]
	2-II	69 [1 CpA]	NT7 [Class C *mec* (*mecA*, *ugpQ*), *dcs & ccrAB2* (IVh)] & ACME-*arc* [1 CpA]	BA000018 [1 CpA]	dt10a [1 CpA]
	2-II	83 [2 Hu]	IV^c^ [Class B *mec* (*mecA*, *ΔmecR1*, *ugpQ*) *dcs* & *ccrAB2*] [1 Hu]	GU235984 [1 Hu]	dt8am [1 Hu]
			NT4 [Class B *mec* (*mecA*, *ΔmecR1*, *ugpQ*) & *ccrAB2*, *ccrAB4*] [1 Hu]	AY786579 [1 Hu]	dt11b [1 Hu]
	2-II	85 [1 Hu]	IV^c^ [Class B *mec* (*mecA*, *ΔmecR1*, *ugpQ*) *dcs* & *ccrAB2*] & ACME-*arc* [1 Hu]	ABSA01000066 [1 Hu]	dt10a [1 Hu]
	2-II	87 [2 Hu]	IV^c^ [Class B *mec* (*mecA*, *ΔmecR1*, *ugpQ*) *dcs* & *ccrAB2*] [2 Hu]	ABSA01000066 [1 Hu]	dt10a [1 Hu]
				BA000018 [1 Hu]	dt10a [1 Hu]
	2-II	125 [1 CpA]	IVg [Class B *mec* (*mecA*, *ΔmecR1*, *ugpQ*) *dcs* & *ccrAB2*] & ACME-*arc* [1]	ABSA01000066 [1 CpA]	dt10a [1 CpA]
	S	539 [1 Hu]	IV^c^ [Class B *mec* (*mecA*, *ΔmecR1*, *ugpQ*) *dcs* & *ccrAB2*] [1 Hu]	AY786579 [1 Hu]	dt10a [1 Hu]
	S	264 [1 Hu]	VI [class B *mec* (*mecA*, *ΔmecR1*, *ugpQ*), *ccrAB4*] [1 Hu]	AB037671 [1 Hu]	dt10a [1 Hu]
	9	490 [1 Hu]	NT5 [*mecA*, *mecI*, *mecR1*, *ugpQ*, *dcs* & *ccrAB1*] [1 Hu]	BA000018 [1 Hu]	dt8f [1 Hu]
	2-I	592 [2 Hu]	IV^c^ [Class B *mec* (*mecA*, *ΔmecR1*, *ugpQ*) *dcs* & *ccrAB2*] [1 Hu]	ABSA01000066 [1 Hu]	dt9bd [1 Hu]
			IVc [Class B *mec* (*mecA*, *ΔmecR1*, *ugpQ*) *dcs* & *ccrAB2*] [1 Hu]	ABSA01000066 [1 Hu]	dt9bd [1 Hu]
**MSSE [6 Hu, 1 CpA]**	2-I	35 [2 Hu]	SCC1 [*ccrAB2*] [2 Hu]	NA [2 Hu]	NA [2 Hu]
	2-II	152 [1 Hu]	None [1 Hu]	NA [1 Hu]	NA [1 Hu]
	2-II	166 [1 CpA]	SCC 2 [*ccrB1* & *ccrAB2*] & ACME-*arc* [1 CpA]	NA [1 CpA]	NA [1 CpA]
	2-II	256 [1 Hu]	SCC1 [*ccrAB2*] [1 Hu]	NA [1 Hu]	NA [1 Hu]
	13	357 [2 Hu]	None [2 Hu]	NA [2 Hu]	NA [2 Hu]
**MRSH [7 Hu, 13 CpA]**	1	1 [5 Hu, 3 CpA]	NT9 [class C *mec (mecA*, *ugpQ)*, *ccrB4*] [4 Hu, 1 CpA]	ABSA01000066 [2 Hu]	dt11v [2 Hu]
				AY786579 [2 Hu, 1CpA]	dt11v [2 Hu], dt11ca [1 CpA]
			NT12 [class B *mec (mecA*, *ΔmecR1*, *ugpQ)*, *ccrAB4*, *ccrA1*, *dcs*] & ACME-*arc* [1 Hu]	AY786579 [1 Hu]	dt10a [1 Hu]
			V_T_ [Class C *mec* (*mecA*, *ugpQ)*, *pls*, *kdp & ccrAA*, *ccrC* (*ccrC2 & ccrC8*)] [1 CpA]	GQ902038 [1 CpA]	dt10a [1 CpA]
			V_T_ [Class C *mec* (*mecA*, *ugpQ) & ccrAA*, *ccrC* (*ccrC2 & ccrC8*)] [1 CpA]	GQ902038 [1 CpA]	dt11cu [1 CpA]
	1	2 [5 CpA]	NT10 [class C *mec (mecA*, *ugpQ*)] [4 CpA]	AY786579 [4 CpA]	dt11a [4 CpA]
			NT11 [class C *mec (mecA*, *ugpQ*), *ccrA3*, *ccrB4*] [1 CpA]	AY786579 [1 CpA]	dt9bd [1 CpA]
	1	3 [2 CpA]	V_T_ [Class C *mec* (*mecA*, *ugpQ) & ccrAA*, *ccrC* (*ccrC2 & ccrC8*)] [2 CpA]	GQ902038 [2 CpA]	dt11a [2 CpA]
	1	4 [1 CpA]	V [Class C *mec* (*mecA*, *ugpQ)*, *pls*, *kdp & ccrAA*, *ccrC* (*ccrC2* only)] [1 CpA]	GQ902038 [1 CpA]	dt11a [1 CpA]
	1	5 [1 CpA]	NT10 [class C *mec (mecA*, *ugpQ*)] [1 CpA]	BA000018 [1 CpA]	dt11a [1 CpA]
	1	6 [1 CpA]	V_T_ [Class C *mec* (*mecA*, *ugpQ) & ccrAA*, *ccrC* (*ccrC2 & ccrC8*)] [1 CpA]	GQ902038 [1 CpA]	dt11a [1 CpA]
	1	8 [1 Hu]	NT9 [class C *mec (mecA*, *ugpQ)*, *ccrB4*] [1 Hu]	AY786579 [1 Hu]	dt11v [1 Hu]
	S	9 [1 Hu]	V [Class C *mec* (*mecA*, *ugpQ) & ccrAA*, *ccrC* (*ccrC2*)] [1 Hu]	GQ902038 [1 Hu]	dt5i [1 Hu]
**MSSH [1 Hu]**	1	1 [1 Hu]	SCC 3 [*ccrAA*, *ccrA4*] [1 Hu]	NA [1 Hu]	NA [1 Hu]

^a^ Sequence types (STs) were determined using species-specific multilocus sequence typing (MLST) schemes as previously described [32, 55].

^b^ Genes commonly associated with SCC*mec* elements were detected using the StaphyType DNA array kit (Alere Technologies GmbH, Jena, Germany). Any isolates found to carry unusual combinations of SCC or SCC*mec* genes using the DNA microarray were further characterized using multiplex PCRs as previously described [44, 45, 50, 52].

^c^ These isolates could not be subtyped by PCR [52] despite harboring *mec* and/or *ccr* genes indicative of SCC*mec* IV.

^d^ MR isolates were subjected to DNA microarray analysis to detect the alleles of *mecA* present as previously described [42]. The *mecA* alleles detected are described according to their GenBank accession numbers.

Abbreviations: Hu, Human; CpA, Companion animal; MR, Methicillin resistance, CC, Clonal complex; ST, Sequence type; SCC, staphylococcal cassette chromosome; dt, *dru* type; MRSE, methicillin-resistant *S*. *epidermidis*; MSSE, methicillin-susceptible *S*. *epidermidis*; MRSH, methicillin-resistant *S*. *haemolyticus*; MSSH, methicillin-susceptible S. *haemolyticus*; ACME, arginine catabolic mobile element; NT, Non-typeable SCC*mec* type; S, singleton; NA, Not applicable.

### Confirmation of isolates as *S*. *epidermidis* and *S*. *haemolyticus*

Isolates were confirmed as either *S*. *epidermidis* or *S*. *haemolyticus* by PCR amplification and sequencing of the 16S rRNA gene using previously described primers [35]. Sequence analysis was performed using the BioNumerics (version 7.1; Applied Maths, Ghent, Belgium), and ApE (v1.17) software packages. Homology searches were performed using BLAST (http://blast.ncbi.nlm.nih.gov/Blast.cgi) [36].

### Antimicrobial susceptibility testing

All isolates were investigated for MR either as described previously [37] using 10 μg and 30 μg cefoxitin disks (Oxoid Ltd., Basingstoke United Kingdom) according to the European Committee on Antimicrobial Susceptibility Testing (EUCAST) methodology and interpretive criteria for disk diffusion tests or using oxacillin broth microdilution assays according to the Clinical Laboratory Standards Institute (CLSI) methodology for broth microdilution [38, 39]. All isolates underwent antimicrobial susceptibility testing against a panel of 23 antimicrobial agents used for antibiogram-resistogram (AR) typing according to EUCAST methodology and a combination of the interpretive criteria by EUCAST [39], CLSI [38] and Rossney *et al*. [37]. The 23 agents tested were amikacin, ampicillin, cadmium acetate, chloramphenicol, ciprofloxacin, erythromycin, ethidium bromide, fusidic acid, gentamicin, kanamycin, lincomycin, mercuric chloride, mupirocin, neomycin, phenyl mercuric acetate, rifampicin, spectinomycin, streptomycin, sulphonamide, tetracycline, tobramycin, trimethoprim, and vancomycin. All isolates were also tested for clindamycin and linezolid resistance using EUCAST methodology and interpretive criteria. All disc concentrations and interpretive criteria used are listed in S2 Table. The EUCAST and CLSI recommended *S*. *aureus* reference strains ATCC29213 and ATCC25923 were used as quality control strains for antimicrobial susceptibility testing. Multidrug-resistance (MDR) was defined as resistance to three or more classes of antimicrobial agents.

### DNA isolation, PCR and sequencing

Total genomic DNA for use in 16S rDNA sequencing, DNA microarray profiling, MLST, SCC*mec* typing and *dru* typing was extracted using the StaphyType kit (Alere Technologies GmbH, Jena, Germany) according to the manufacturer’s instructions. Apart from PCR for DNA microarray profiling, all PCRs were performed using GoTaq DNA polymerase (Promega, WI, USA). PCR products were purified using the GenElute PCR clean-up kit (Sigma, Wicklow, Republic of Ireland) or, for MLST, the QIAquick 96 well PCR purification kit (Qiagen, Crawley, UK). All DNA sequencing reactions were carried out commercially by Source BioScience LifeSciences (Waterford, Republic of Ireland).

### DNA microarray profiling

All isolates underwent DNA microarray profiling using the StaphyType kit (Alere) according to the manufacturer’s instructions. The DNA microarray detects 333 gene targets including staphylococcal antimicrobial-resistance, virulence, SCC*mec* and ACME-*arc* genes [40, 41]. All isolates harboring the *mecA* gene were subjected to additional DNA microarray profiling using separate *mecA* allele typing arrays (Alere) designed to identify 15 different *mecA* alleles as previously described [42]. Using this method, the *mecA* alleles were designated according to their GenBank accession numbers [42]. The sequences of *mecA* alleles were compared using the GenBank sequences and the alignment software program Mega 6.0 [43].

### SCC*mec* typing

Any isolates found to carry unusual combinations of SCC or SCC*mec* genes using the StaphyType DNA microarray underwent multiplex PCRs to confirm the presence or absence of particular genes. This included previously described multiplex SCC*mec* typing PCRs to detect the *mec* gene complexes A-C, the *ccr* gene complexes *ccrAB1*-*AB4* and *ccrC* and the joining regions of SCC*mec* types I-IV [44, 45]. The following *S*. *aureus* strains were used as positive controls for SCC*mec* typing PCRs: phenotype II 43.2 (SCC*mec* I, *ccrAB1*) [46], CA05 (SCC*mec* IV, class B *mec*, *ccrAB2*) [47], WIS (class C *mec*) [48], 07.4/0237 (SCC*mec* II) [46], JCSC 4744 (IVA) [44], M00/0005.2 (*ccrAB4*) [49], and E0898 (SCC*mec* III, class A *mec*, *ccrAB3 ccrC*) [49]. All isolates found to carry *ccrC* underwent multiplex PCR for the *ccrC* allotypes *ccrC2* and *ccrC8* to differentiate between SCC*mec* V (*ccrC2*) and V_T_ (*ccrC2* and *ccrC8*), as described previously [50], using the *S*. *aureus* clinical isolate M06/0318 (SCC*mec* V_T_) as a positive control strain [51]. All isolates found to harbor SCC*mec* IV or possible novel SCC*mec* types with *mec* and/or *ccr* genes indicative of SCC*mec* IV, underwent SCC*mec* IV subtyping PCR as previously described [52], using the following *S*. *aureus* strains as positive controls: CA05 (SCC*mec* IVa) [47], 8/63P (SCC*mec* IVb) [47], JCSC4788 (SCC*mec* IVc) [53], JCSC4469 (SCC*mec* IVd) [53], M04/0177 (SCC*mec* IVg) [49] and E1749 (SCC*mec* IVh) [49].

### PCR-based detection of antimicrobial resistance genes

Isolates were subjected to PCR-based detection of antimicrobial resistance genes to confirm (i) the absence of a resistance gene(s) if an isolate exhibited phenotypic resistance to an antimicrobial agent and no corresponding resistance gene was detected using the StaphyType DNA microarray, or (ii) the presence of a resistance gene(s) detected in an isolate using the DNA microarray which did not exhibit phenotypic resistance to the corresponding antimicrobial agent(s). This included PCRs to detect the presence of *aacA-aphD*, *aadD*, *aphA3*, *cat-pC221*, *dfrS1*, *erm*(A), *erm*(C), *ileS2*, *merA*, *merB*, *qacA* and *qacC*. Lastly, PCRs were also performed to detect additional trimethoprim resistance genes (*dfrG* and *dfrK*) not detected using the DNA microarray in isolates that exhibited phenotypic resistance to trimethoprim but lacked *dfrS1*. The oligonucleotide primers used for these PCRs are detailed in S3 Table.

### Direct repeat unit (*dru*) typing

All methicillin-resistant staphylococcal isolates investigated (*n* = 55) were subjected to *dru* typing using previously described primers and thermal cycling conditions [16]. The BioNumerics tandem-repeat sequence typing (TRST) plug-in was used for *dru* sequence analysis and assignment of *dru* types (dts). The *dru* region of five MRSE isolates could not be amplified by the originally described *dru* typing primers. For these isolates, the *dru* region was amplified using previously described primers mecAF and ISmecR that extend from *mecA* to IS*431* (S3 Table) [46]. Minimum spanning trees (MSTs) were constructed based on the dts identified as previously described [54]. Due to the increased likelihood of recombination amongst *S*. *epidermidis* and *S*. *haemolyticus* populations, the bin distance was set to 1%, i.e., the distance between two entries with >99% similarity was 0 (a distance interval of 99 to 100% similarity equals a distance of 0) on the MST, and the distance between two entries with 98 to 99% similarity was 1 (a distance interval of 98 to 99% similarity equals a distance of 1).

### MLST

All isolates were subjected to MLST. A previously described species-specific scheme, including primers and thermal cycling conditions, was used for MLST of *S*. *epidermidis* isolates [55]. A *S*. *haemolyticus*-specific scheme was used for MLST of *S*. *haemolyticus* isolates [32] but primer SH1200R was substituted with a novel primer (SH1200R2 5′-ACCAGGCTTGTCACCATGA-3′) and SH1431F was substituted with a novel primer (SH1431F2 5′-TCAGACCAACAATTCCCACC -3′) to increase amplimer yields. For *S*. *haemolyticus* isolates, thermal cycling conditions consisted of an initial denaturation step of 94°C for two min, followed by 35 repeated cycles of 94°C for one min, 51°C for 30 s and 72°C for 30 s, and a final elongation step of 72°C for five min. Sequence analysis was performed using the ABI prism Seqscape (version 2.6, Applied Biosystems, Foster City, CA) or BioNumerics software. *Staphylococcus epidermidis* alleles and sequence types (STs) were identified using the *S*. *epidermidis*-specific MLST database (http://sepidermidis.mlst.net/) [56]. As there is no publicly available *S*. *haemolyticus* MLST database, alleles and STs were assigned identification numbers using our own in-house database (S4 and S5 Tables). For both species, assignment of STs to CCs was performed using the eBURST algorithm, where an ST was only assigned to a CC if it shared at least 6/7 MLST loci with at least one other ST within a CC [57].

### Statistical analyses

In order to determine if the differences in the prevalence of antimicrobial resistance genes and phenotypes and ACME were significant between SH and SE isolates or between isolates recovered from Hu and CpA hosts, two-tailed Fisher’s exact tests were utilized. These analyses were carried out using GraphPad QuickCalcs (http://www.graphpad.com/quickcalcs/index.cfm).

### Nucleotide accession numbers

The nucleotide sequences of the *mecA*-IS431*mec* amplimers for MRSE isolates 23767, 28427, 31169, 408 996.1, and BM11 that lacked the *dru* region have been deposited in the GenBank database under accession numbers KP265311, KP265312, KP265313, KP265314 and KP265315, respectively.

## Results

### Methicillin resistance, genotypes and SCC-associated genes among SE isolates

In total, 33/40 (82.5%) SE isolates exhibited MR and carried *mecA* (Table 1). Twelve STs were identified amongst the MRSE isolates with 22/33 (66.7%) and 8/33 (24.2%) isolates belonging to CC2 clusters I and II, respectively (Table 1) [33]. ST2 was common to both MRSE-Hu and-CpA isolates, and this was the predominant ST identified amongst both groups (9/22, 40.9% MRSE-Hu and 9/11, 81.8% MRSE-CpA) (Table 1).

Overall 25/33 (75.8%) MRSE isolates were assigned to SCC*mec* types III, IV and VI but a single SCC*mec* type, SCC*mec* IV (most commonly subtype IVc), predominated amongst both MRSE-Hu (13/22, 59.1%) and MRSE-CpA (7/11, 63.6%) (Table 1). Based on DNA microarray analysis and PCR, non-typeable (NT) SCC*mec* elements, tentatively designated NTs 1–8, were detected among 8/33 (24.2%) MRSE (Hu and CpA) isolates, as these lacked, contained additional, or had unusual combinations of *mec* and/or *ccr* genes (Table 1). However, half of these NT SCC*mec* elements consisted of class B *mec* with *ccrAB2* indicative of SCC*mec* IV but they also carried additional *ccr* genes (NTs 1–4, Table 1) with NTs 1–3 also harboring ACME-*arc* (Table 1). Three further NTs carried class A *mec* with unusual combinations of *ccr* genes or ACME-*arc* genes (NTs 5, 6 & 8, Table 1). The final NT SCC*mec* element carried class C *mec* with *ccrAB2*, SCC*mec* IV subtype IVh and ACME-*arc* genes (NT7, Table 1).

Six and four *mecA* alleles were identified amongst the MRSE-Hu and-CpA isolates, respectively (Table 1), but these shared >99.85% DNA sequence identity and differed by a maximum of three nucleotide bases. All *mecA* alleles detected amongst the MRSE-CpA were also detected amongst the MRSE-Hu (Table 1). The *mecA* allele ABSA010000166 previously detected in *S*. *aureus*, *S*. *pseudintermedius* and SE was detected in 10/22 (45.5%) MRSE-Hu and 4/11 (36.4%) MRSE-CpA (Table 1). With the exception of one MRSE-CpA isolate harboring SCC*mec* III, all of the MRSE isolates in which this allele was detected harbored SCC*mec* IV.

Four *mecA* alleles were detected among isolates of the most prevalent MRSE genotype (ST2-MRSE-IVc) with only one allele (ABSA01000066) common to both hosts (6/9, 66.7% MRSE-Hu and 2/9, 22.2% MRSE-CpA; Table 1), however, all *mecA* alleles identified among ST2-SCC*mec* IVc isolates differed by a maximum of three nucleotides indicating their close similarity. The ST2-MRSE-IVc isolates either lacked the *dru* region or were assigned to one of four dts, with non-typeable dts common to Hu and CpA isolates (Table 1). However, two of the remaining ST2-MRSE-IVc dts, dt10h and dt9g, were deemed to be closely related (MST value of 2.5 i.e. 96.5–97% DNA sequence identity; S1A Fig) and were identified from a Hu and CpA host, respectively.

Among the MSSE, four of five STs identified belonged to CC2 (Table 1). Only one MSSE-CpA isolate was identified and was distinct from the Hu isolates in terms of ST and the presence of ACME-*arc* genes. Among the MSSE, two possible novel SCCs (tentatively designated SCCs 1 and 2) were detected consisting of *ccrAB2* alone or in combination with *ccrAB1* and ACME-*arc* genes (Table 1).

The ACME-*arc* genes were more common amongst SE-CpA (6/12, 50%) than SE-Hu (4/28, 14.3%) (*p* = 0.04) (Table 1).

### Methicillin resistance, genotypes and SCC-associated genes among SH isolates

Among the SH isolates 95.2% (20/21) were MR and carried *mecA* (Table 1). Eight STs were identified, seven of which were assigned to a single CC (CC1) (Table 1 and S4 Table). While ST1 was the most common ST among SH isolates and was the only ST identified in both SH-Hu and-CpA isolates, ST2 was more common among the SH-CpA isolates (Table 1).

Previously described SCC*mec* elements, either SCC*mec* V or V_T_, were detected in only 7/20 (35%) MRSH isolates (Table 1). Four NT SCC*mec* elements were detected and tentatively described as NTs 9–12. With the exception of NT12, all MRSH SCC*mec* NTs harbored class C *mec* and various combinations of *ccr* genes (Table 1). According to microarray analysis, the NT12 isolate carried class B *mec* and *ccrAB2* indicative of SCC*mec* IV, as well as *ccrA1* and was the only SH isolate that harbored the ACME-*arc* genes. No SCC*mec* IV subtype was identified by PCR. Multiplex SCC*mec* typing PCR and sequencing revealed that this isolate harbored *ccrAB4*, with 100% DNA sequence identity to *ccrAB4* in *S*. *haemolyticus* (GenBank accession no. AB587081.1) rather than *ccrAB2* [58]. This SH *ccrAB4* allele exhibited 91% and 87% DNA sequence identity to *ccrAB4* and *ccrAB2* in *S*. *aureus*, respectively, which the array *ccr* primers and probes are based on. The ambiguity in the identification of the *ccrAB* alleles in this SH isolate using the DNA microarray may be linked to this.

Possible novel SCC*mec* V and V_T_ subtypes were detected in two additional MRSH-CpA isolates which carried the *kdp* and *pls* genes in addition to the class C *mec* and *ccrAA* and *ccrC* genes (Table 1).

Three *mecA* alleles were identified among both the MRSH-Hu and-CpA isolates investigated, which shared >99.9% DNA sequence identity and differed by a maximum of two nucleotide bases indicating their close similarity. The *mecA* alleles AY786579 and GQ92039 were detected in 10/20 (50%) and 7/20 (35.0%) MRSH, respectively, both being detected in MRSH-Hu and–CpA isolates (Table 1).

ST1-MRSH-NT9 was the only common ST and SCC*mec* type combination detected among both MRSH-Hu (*n* = 4) and-CpA (*n* = 1). Two *mecA* alleles and two dts were detected amongst these five isolates, with only one *mecA* allele common to Hu and CpA isolates (Table 1). However, the *mecA* alleles (ABSA01000066 & AY786579; one nucleotide difference) and dts (dt11v & dt11ca; MST value of 2 i.e. 97–98% similarity, S1B Fig) were closely related. All of the other ST and SCC*mec* type combinations were unique to either MRSH-Hu or MRSH-CpA. The SCC*mec* types V or V_T_ were detected in MRSH-CpA (*n* = 6) and MRSH-Hu (*n* = 1), but these isolates were assigned to CC1 and as a singleton, respectively, and were assigned to four distinct dts (Table 1 and S1 Fig). However, all SCC*mec* types V or V_T_ isolates harbored the GQ902038 *mecA* allele.

Only one MSSH isolate was identified (Hu). This isolate was identified as ST1 and harbored a NT SCC element, consisting of *ccrAA* and *ccrA4* (SCC3, Table 1).

### Antimicrobial susceptibility

The antimicrobial resistance phenotypes and genes detected among the isolates investigated are shown in Table 2 and S1 Table. Multidrug resistance (MDR) was detected among 96.7% (59/61) of isolates and resistance to almost all the classes of antimicrobial agents investigated was detected among both the SE and SH isolates.

**Table 2:** Prevalence of antimicrobial resistance genes and phenotypic resistance to antimicrobial agents among *S*. *epidermidis* and *S*. *haemolyticus* isolates from humans and companion animals^a^.

Class of antimicrobial agents	Resistance gene detected	Relevant resistance phenotype detected^a^	No. of isolates (%)			
			SE-Hu (*n* = 28)	SE-CpA (*n* = 12)	SH-Hu (n = 8)	SH-CpA (n = 13)
**Aminoglycosides**	*aacA-aphD* ^b^	Ak, Gn, Kn, Tb^b^	12 (42.9)	8 (66.7)	7 (75)	12 (92)
	*aadD* ^b^	Ak, Kn, Nn, Tb^b^	2 (7.1)	4 (33.3)	2 (25)	6 (46.2)
	*aphA3*	Kn, Nm	0 (0)	3 (25)	1 (12.5)	6 (46.2)
	N/A^c^	Sp, St	0 (0)	0 (0)	0 (0)	0 (0)
**Antiseptics, disinfectants and intercalating dyes**	*qacA* ^*d*^	Eb^*d*^	23 (82.1)	9 (75)	5 (62.5)	7 (53.8)
	*qacC* ^*d*^	Eb^*d*^	3 (10.7)	0 (0)	1 (12.5)	2 (15.4)
**Beta-lactams (excluding methicillin)**	*blaZ*	Ap	28 (100)	11 (91.7)	7 (87.5)	13 (100)
**Chloramphenicol**	*cat*-pC221	Cl	0 (0)	0 (0)	0 (0)	5 (38.4)
	*fexA*	Cl	0 (0)	2 (16.7)	0 (0)	0 (0)
**Fluoroquinolones**	N/A^c^	Cp	16 (57.1)	10 (83.3)	6 (75)	12 (82.3)
**Fusidic acid**	*fusB* ^e^	Fd^e^	18 (64.3)	11 (91.7)	0 (0)	9 (69.2)
	*fusC*	Fd	3 (10.7)	0 (0)	1 (12.5)	0 (0)
**Glycopeptides**	*vanA*, *B*, *Z*	Vn	0 (0)	0 (0)	0 (0)	0 (0)
**Lincosamides**	*lnu*(A)	Da & Ln	0 (0)	0 (0)	0 (0)	2 (15.4)
**Lincosamides, pleuromutilins and streptogramin A/B compounds**	*vga*	Da & Ln	2 (7.1)	0 (0)	0 (0)	4 (30.8)
	*vga*(A)	Da & Ln	2 (7.1)	0 (0)	0 (0)	0 (0)
	*vga*(B)	Da & Ln	0 (0)	0 (0)	0 (0)	0 (0)
**Linezolid**	*cfr*	Lz	0 (0)	0 (0)	0 (0)	0 (0)
**Macrolides**	*msr*(A)	Er	13 (46.4)	6 (50)	7 (87.5)	12 (92.3)
	*mph*(C)	Er	5 (17.9)	1 (8.3)	7 (87.5)	12 (92.3)
**Macrolides lincosamides & streptogramin B compounds**	*erm*(A)	Da, Er & Ln	2 (7.1)	0 (0)	0 (0)	0 (0)
	*erm*(B)	Da, Er & Ln	0 (0)	1 (8.3)	0 (0)	1 (7.7)
	*erm*(C)^f^	Da, Er & Ln	9 (32.1)	8 (66.7)	2 (25)	4 (30.8)
**Mercury**	*merA* & *merB*	Mc, Pma	4 (14.3)	2 (16.7)	0 (0)	0 (0)
**Mupirocin**	*ileS2*	Mp	9 (32.1)	1 (8.3)	0 (0)	1 (7.7)
**Rifampicin**	N/A^d^	Rf	21 (75.0)	10 (83.3)	2 (25)	12 (92.3)
**Streptogramin A compounds**	*vat*(B)	Ln	2 (7.1)	0 (0)	0 (0)	0 (0)
**Sulphonamide**	N/A^c^	Su	21 (75.0)	8 (66.7)	7 (87.5)	11 (84.6)
**Tetracycline**	*tet*(M)	Te	0 (0)	0 (0)	0 (0)	3 (23.1)
	*tet*(K)	Te	2 (7.1)	5 (41.7)	0 (0)	10 (76.9)
**Trimethoprim**	*dfrS1* ^*g*^	Tp	24 (85.7)	9 (75)	2 (25)	1 (7.7)
	*dfrG*	Tp	1 (3.6)	0 (0)	7 (87.5)	9 (69.2)
	*dfrK*	Tp	1 (3.6)	2 (16.7)	7 (87.5)	9 (69.2)
	Total no. MDR^h^		27	12	7	13

^a^Full resistance profiles for all isolates are shown in S1 Table. Antimicrobial resistance patterns were determined by testing the susceptibility of isolates to a panel of 25 antimicrobial agents including amikacin (Ak), ampicillin (Ap), cadimium acetate (Cd), chloramphenicol (Cl), ciprofloxacin (Cp), clindamycin (Da), ethidium bromide (Eb), erythromycin (Er), fusidic acid (Fd), gentamicin (Gn), kanamycin (Kn), lincomycin (Ln), linezolid (Lz), mercuric chloride (Mc), mupirocin (Mp), neomycin (Nm), phenyl mercuric acetate (Pma), rifampicin (Rf), spectinomycin (Sp), streptomycin (St), sulphonamide (Su), tetracycline (Te), tobramycin (Tb), trimethoprim (Tp) and vancomycin (Vn).

^b^Not all isolates harboring the *aadD* or *aphA3* genes exhibited phenotypic resistance to all of the relevant aminoglycosides. Of the 40 isolates harboring *aacA-aphD*, only five exhibited amikacin resistance. The *aadD* gene was detected in 14 isolates, four of which were amikacin-resistant; three of these 14 isolates exhibited only kanamycin and tobramycin resistance.

^c^N/A, not applicable as resistance to each of these agents is mediated by mutations, or by genes not detected by the DNA microarray. The presence of these mutations or genes were not determined in these isolates in the present study.

^d^ Ten of the isolates harboring *qacA* and two of the isolates harboring *qacC* exhibited susceptibility to quaternary ammonium compounds.

^e^The *fusB* gene was detected in one isolate which lacked the appropriate resistance phenotype.

^f^Of the 23 isolates harboring *erm*(C), all exhibited erythromycin resistance, however 13 of these isolates were susceptible to lincomycin.

^g^The *dfrS1* gene was detected in eight isolates which lacked the appropriate resistance phenotype.

^h^MDR, Multidrug-resistance, defined as resistance to three or more classes of antimicrobial agents.

However, differences were identified in the prevalence of phenotypic antimicrobial resistance and resistance genes detected among Hu and CpA isolates and SE and SH isolates. These differences are described below in more detail.

### Comparison of antimicrobial resistance genes amongst SE and SH isolates

Genes encoding resistance to fusidic acid and trimethoprim, primarily encoded by *fusB* and *dfrS1*, respectively, were significantly (*p* < 0.05) more prevalent among SE than SH isolates (Table 2). In contrast, resistance to macrolides encoded by *msr*(A) and *mph*(C), and aminoglycosides, encoded by *aacA-aphD* and *aphA3* were significantly (*p* < 0.05) more common among the SH isolates (Table 2). The trimethoprim resistance genes *dfrG* and *dfrK* were significantly more prevalent (*p* = 0.0001) amongst SH than SE isolates (Table 2). Although tetracycline resistance encoded by *tet*(K) was detected among both SH and SE isolates, *tet*(M) was only detected among the SH isolates (*n* = 3).

### Comparison of antimicrobial resistance genes amongst isolates from Hu and CpA hosts

Resistance to aminoglycosides encoded by *aadD* and *aphA3*, tetracycline encoded by *tet*(K) and fusidic acid encoded by *fusB*, were significantly (*p* < 0.05) more common among the CpA than Hu isolates (Table 2). Resistance to ciprofloxacin and rifampicin was also significantly more common in the CpA isolates (*p* < 0.05). Resistance to chloramphenicol was detected in CpA isolates only (*p* = 0.001) where it was encoded by *fexA* in SE-CpA isolates and *cat*-pC221 among SH-CpA isolates (Table 2).

## Discussion

Both similarities and differences were detected in the genotypes, SCC/SCC*mec* associated genes, *mecA* alleles and dts amongst both MRSE and MRSH isolates from Hu and CpA infections. A single ST (ST2) was identified among 45% of both SE-Hu and SE-CpA isolates. While ST1 was detected among both SH-Hu and SH-CpA isolates, 75% of Hu isolates and only 23.1% of CpA isolates were identified as ST1, whereas ST2 was identified amongst 38.5% of CpA isolates and was not detected in Hu isolates. However, it is important to note that almost all STs identified within each staphylococcal species belonged to a single CC and therefore isolates within these STs are clonally related, including the SH STs 1 and 2 which differed by just two MLST alleles (S4 Table). Furthermore, there was an association between SCC*mec* types and the different staphylococcal species, as SCC*mec* IV (or components thereof) was detected amongst 75.8% of MRSE and SCC*mec* V/V_T_ (or components thereof) was detected amongst 95% of MRSH investigated. These findings further highlight the genetic similarity that exists between SE and SH isolates recovered from Hu and CpA hosts.

The *mecA* allele and *dru* typing enhanced discrimination of isolates with the same ST and SCC*mec* type. However, several MRSE and MRSH–Hu and-CpA isolates with the same ST and SCC*mec* type but with different *mecA* alleles or dts were still deemed to be closely related due to a high degree of sequence identity in the *dru* and *mecA* sequences (Table 1 and S1 Fig). The *dru* and *mecA* sequence variation demonstrates that minor genetic variation exists amongst MRSE and MRSH isolates recovered from-Hu and-CpA hosts, despite the overall species-specific similarities that exist with regard to clonal lineages and SCC*mec* types. The use of MSTs and the comparison of the DNA sequence identity among *mecA* alleles is particularly important in this study as variation may have accumulated within these regions in epidemiologically distinct but genetically related isolates over time. However, the accumulation of variation in *mecA* and *dru* in MRSE and MRSH requires further investigation. In the present study, five ST2-SCC*mec* IVc MRSE isolates (both Hu and CpA) lacked the *dru* region. This has been reported previously in *S*. *epidermidis* and *S*. *aureus*, albeit infrequently [59, 60]. It will be important to determine how widespread the absence of a *dru* region is in each of the staphylococcal species before it is more widely used for investigating these species. The highly clonal nature of the SH population is reflected by identification of closely related STs within a single CC and a limited number of dts. More informative methods such as whole-genome sequencing should be used to enhance discrimination of SH isolates.

Similar to previous reports, the present study revealed an enrichment of specific SCC*mec* types and genes in association with both MRSE and MRSH [13, 22, 61], and yet extensive genetic diversity within some of these SCC*mec* types. Eight distinct NT SCC*mec* elements were identified among eight MRSE isolates, some of which are similar to previously described NTs in MRSE [5, 61]. It is difficult to determine if the NTs identified in the present study are identical to those described previously due to the different SCC*mec* typing methods used and the precise genetic organization of these NTs have not been fully determined in either the present or previous studies. This finding correlates with recent research that revealed NT SCC*mec* elements in 21.3% of MRSE from livestock, farmers and hospital-associated MRSE [5]. In addition, three NTs were identified among the MRSH, one of which (NT10) is similar to a not fully characterized NT previously detected in MRSH [5]. It is important to emphasize that the genetic organization of the currently recognized SCC*mec* elements I-XI in staphylococci is based on complete nucleotide sequencing of the regions concerned [62–64]. The genetic organization of the NT SCC*mec* elements identified in this study are currently being investigated by whole-genome sequencing in order to definitively establish their exact relationships to SCC*mec* elements I-XI.

The *mecA* allele typing provided further evidence of the specific SCC*mec* genes within individual CoNS species. Although the most prevalent *mecA* allele (AY786579) was common to MRSE and MRSH, alleles BA000018 and AB037671, (both previously detected in *S*. *aureus* [42]) were only detected among MRSE and allele GQ902038 (previously identified *S*. *aureus*, *S*. *haemolyticus* and *S*. *pseudintermedius* [42]) was only detected among MRSH (Table 1) further highlighting the spread of *mecA* among staphylococcal species.

The ACME-*arc* genes were more common amongst SE isolates and were only detected in one SH-Hu isolate. The latter finding correlates with previous whole-genome sequence analysis of 134 SH isolates from nosocomial infections, which revealed a low prevalence of ACME *arcA* [65]. Other studies suggested that ACME originated in *S*. *haemolyticus* [66], although the findings of the present and previous study [65] do not support this. Interestingly, among the MRSE isolates, ACME was more common among CpA (50%) than Hu (17.9%) isolates indicating a possible reservoir for ACME among CpA isolates.

Differences were identified among isolates from Hu and CpA hosts in terms of specific antimicrobial resistance genes and phenotypes. For example, the prevalence of chloramphenicol (encoded by *cat-pC221* in SH-CpA and *fexA* gene in SE-CpA), ciprofloxacin, rifampicin, tetracycline, fusidic acid and aminoglycoside resistance was significantly higher in CpA isolates (*p* < 0.05). In contrast, a recent study revealed that resistance to rifampicin, ciprofloxacin and fusidic acid was more common among hospital-associated SE isolates than among isolates from animals, although the animals investigated were all livestock [5]. As many of these drugs are used in both veterinary and human medicine, the transmission of resistant CoNS between humans and companion animals is clinically important, particularly as levels of pet ownership have increased over recent decades [67]. The increased prevalence of tetracycline and chloramphenicol resistance in CpA isolates may reflect the different ecological niches within different hosts and different selective pressures due to variations in common prescription practices between human and veterinary medicine as well as the overall use of antimicrobials in veterinary medicine [68]. However, it is important to note that all CpA isolates investigated were from the diagnostic laboratory of a tertiary referral veterinary hospital. Animals attending such a hospital in many instances would have received previous antimicrobial treatment from the referral practices.

The results of this study suggest that similar to SCC*mec* types and genes, SE and SH isolates are a reservoir for antimicrobial resistance genes, and in some instances, individual resistance genes are significantly more common among either species. To our knowledge, this study is the first to highlight both SE and SH species- and host- specific significant differences in the prevalence of particular antimicrobial resistance genes and phenotypes, suggestive of specific contributions of these staphylococcal species from different hosts to the staphylococcal gene pool. The trimethoprim resistance gene *dfrS1* and the fusidic acid resistance gene *fusB* were significantly more common among SE (*p <*0.05), whereas the trimethoprim resistance genes *dfrG* and *dfrK*, the aminoglycoside resistance genes *aacA-aphD* and *aphA3* and the macrolide resistance genes *msr*(A) and *mph*(C) were significantly more common among SH (*p* < 0.05), the latter of which is in agreement with previous studies [22].

This study has revealed that despite being epidemiologically unrelated, the populations of SE and SH isolates recovered from infections in both Hu and CpA hosts belong to the same clonal lineages and harbor similar SCC*mec* genes. The findings of the present study suggest that, even in the absence of direct transmission, populations of both SE and SH with a shared genetic background are capable of causing infections in Hu and CpA hosts. Host and species-specific characteristics were also identified in relation to antimicrobial resistance genes and phenotypes, SCC*mec* and ACME. We have highlighted significant differences in the prevalence of the specific genes encoding resistance to fusidic acid, aminoglycosides, macrolides and trimethoprim amongst SE and SH isolates, and we have shown that SE and SH isolates from CpA hosts may constitute a reservoir for ACME and genes encoding resistance to multiple antimicrobial agents including aminoglycosides, tetracycline, fusidic acid and chloramphenicol. Lastly, *dru* and *mecA* allele typing were found to be a useful addition to MLST and SCC*mec* typing for differentiating closely related isolates, but dts needs to be carefully considered in longer-term studies so that similarities are not overlooked.

## Supporting Information

S1 Fig Minimum spanning trees (MSTs) of *dru* types. (XLSX)

S1 Table Summary of isolate data. (XLSX)

S2 Table Antimicrobial agents and breakpoints. (XLSX)

S3 Table Oligonucleotide primer sequences. (XLSX)

S4 Table Allelic profiles identified by *Staphylococcus haemolyticus* MLST. (XLSX)

S5 Table Allele sequences identified by *Staphylococcus haemolyticus* MLST. (XLSX)
